# Improvement in the management of gout is vital and overdue: an audit from a UK primary care medical practice

**DOI:** 10.1186/1471-2296-14-170

**Published:** 2013-11-14

**Authors:** Elizabeth Cottrell, Valerie Crabtree, John J Edwards, Edward Roddy

**Affiliations:** 1 Research Institute for Primary Care & Health Sciences, Keele University, Keele, ST5 5BG, Staffordshire

**Keywords:** Gout, Management, Audit, Primary care, Allopurinol, Serum uric acid

## Abstract

**Background:**

Gout is estimated to affect 1.4% of adults in the UK. Appropriate and timely management is essential to reduce the risk of further flares, complications, and to reduce cardiovascular disease risk. The British Society for Rheumatology and British Health Professionals in Rheumatology (BSR/BHPR) and the European League Against Rheumatism (EULAR) have published guidance regarding the management of gout, thereby providing standards against which performance can be measured. This audit was designed to assess the extent to which patients diagnosed with gout in one primary care medical practice in North Staffordshire, UK, are managed in accordance with current best practice guidelines, and to identify strategies for improvement where appropriate.

**Methods:**

Audit criteria were derived from the EULAR and BSR/BHPR guidelines; standards were set arbitrarily, but with consideration of patient comorbidity and other factors which may influence concordance. An electronic search of the practice records was performed to identify adults with a diagnosis of gout. Medical record review with a descriptive analysis was undertaken to assess the extent to which medical management adhered to the predefined standards.

**Results:**

Of the total ≥18 year-old practice population (n = 8686), 305 (3%) patient records included a diagnosis of gout. Of these, 74% (n = 226) had an electronic record of serum uric acid (SUA), and 11% (n = 34) and 53% (n = 162) a measure of estimated glomerular filtration rate (eGFR) ever and serum glucose since diagnosis respectively. 34% (n = 105) of patients had ever taken urate-lowering therapy with 25% (n = 77) currently prescribed this at the time of data extraction. Dose adjustment and monitoring of treatment according to SUA was found to be inadequate. Provision of lifestyle advice and consideration of comorbidities was also lacking.

**Conclusions:**

The primary care management of gout in this practice was not concordant with national and international guidance, a finding consistent with previous studies. This demonstrates that the provision of guidelines alone is not sufficient to improve the quality of gout management and we identify possible strategies to increase guideline adherence.

## Background

The estimated prevalence of gout amongst the UK population is 1.4%. The UK annual primary care consultation prevalence among adults ≥18 years is between 4.2/1000 and 4.9/1000 [1]. Prevalence increases with age such that 7% of men aged 75-84 yr are affected [2]. Timely and effective treatment of gout is necessary to reduce the risk of further flares, chronic polyarthritis and tophaceous disease [3].

Current guidelines by the British Society for Rheumatology and British Health Professionals in Rheumatology (BSR/BHPR) [3] and the European League Against Rheumatism (EULAR) [4], relating to the management of gout in primary care are similar and may be used by primary care health professionals in the UK. Both guidelines encourage urate-lowering therapy (ULT) if patients have two or more attacks of acute gout, or have other risk factors that would make further attacks likely. In such instances ULT should be prescribed and titrated according to the serum uric acid (SUA). The EULAR guidelines suggest a target SUA of ≤360 μmol/l while the BSR/BHPR guidelines advocate a lower target (SUA ≤300 μmol/l). Both EULAR and BSR/BHPR suggest commencing allopurinol at a dose of 100 mg/day (BSR/BPHPR 50-100 mg/day) and increasing by 100 mg (50-100 mg) every few weeks according to SUA and renal function [3,4]. The systemic inflammatory response in acute gout commonly leads to a transient reduction in SUA [5], therefore BSR/BPHPR guidelines suggest SUA is checked 4–6 weeks post-flare [3].

In addition to ULT, guidelines recommend that patients should be given lifestyle advice where appropriate to modify risk factors for hyperuricaemia and gout. Guidelines recommend: optimising weight and dietary modifications, particularly restricting the intake of purine-rich foods and limiting alcohol consumption [3,4]. Intervention studies show that weight loss and restriction of dietary purines have modest urate-lowering effects [6,7].

The primary care management of gout has previously been examined. In 2000, prior to the publication of the current BSR/BHPR and EULAR guidelines, Pal et al. [8] undertook an audit of 74,111 patients from 12 practices in the UK. Following the publication of the EULAR recommendations for the management of gout in 2007, Roddy et al. used a questionnaire survey to study the management of primary care patients in two general practices in the UK [9]. Further information from the USA was gathered by Wall et al. [10] who undertook a review of medical records of patients seen with gout between 2004–7 to compare care against guideline recommendations. Common to all previous findings is that many patients have an inadequately controlled SUA level and have not received or retained adequate lifestyle information [8-10].

The aim of this audit was to extend assessment of practice beyond the scope of previous work so as to include a comprehensive assessment of the approaches to lifestyle modification and medical management of gout itself, and to assess the extent to which such management is in line with current best practice guidelines. The wider implications of this diagnosis on the patient’s overall risk of morbidity and mortality will also be addressed through consideration of associated comorbidities and cardiovascular risks.

## Methods

### Standards, criteria and targets

Audit criteria were derived from the EULAR and BSR/BHPR guidelines. Standards were set following consideration of guideline recommendations and patient comorbidities. Audit criteria and standards are outlined in Table 1.

**Table 1:** Standards, criteria and targets used in gout audit

**Theme of care**	**Guideline statement**	**Criteria**	**Standards**
Assessment of gout patients	All patients presenting with acute gout should have SUA, renal function and glucose assessed 4-6wk after acute attack [3]	1.1 Adult patients with a diagnosis of gout have ever had a recorded SUA	≥90%
		1.2 Adult patients with a diagnosis of gout have ever had a recorded eGFR	≥90%
		1.3 Adult patients with a diagnosis of gout have had a recorded serum glucose since diagnosis	≥90%
Management of recurrent gout	The therapeutic goal of urate lowering therapy…is achieved by maintaining the SUA below the saturation point for urate (≤360 μmol/l) [4]	2.1 Adult patients currently prescribed (prescription in the last 2 months) allopurinol have a SUA measured in the last year	≥90%
		2.2 Adult patients currently prescribed (prescription in the last 2 months) allopurinol have an SUA ≤ 360 μmol/l	≥80%
Monitoring of gout patients	Annual measurements of plasma renal function and SUA [3]	3.1 Adult patients with a diagnosis of gout have had a SUA in the last year	≥90%
		3.2 Adult patients with a diagnosis of gout have had a eGFR measured in the last year	≥90%
Lifestyle advice	All patients with gout should be given advice about diet, alcohol restriction, weight management and fluid intake.[3,4]	4.1 Adult patients with a diagnosis of gout with evidence of advice regarding diet recorded	≥90%
	[It is recognised that such information may be provided in written format in a patient information leaflet]	4.2 Adult patients with a diagnosis of gout with evidence of advice regarding alcohol recorded	≥90%
		4.3 Adult patients with a diagnosis of gout with evidence of advice regarding fluid intake recorded	≥90%
		4.4 Adult patients with a diagnosis of gout have a record of BMI	≥90%
Addressing comorbidities	Associated comorbidities and cardiovascular risk factors should be addressed as an important part of the management of gout [4]	5.1 Adult patients with a diagnosis of gout have a recorded CVD risk assessment score	≥90%
		5.2 Adult patients with a diagnosis of gout and a 10 yr risk of CVD ≥20% are prescribed lipid lowering therapy	≥75%
	Diuretic therapy should be stopped if possible in patients with a diagnosis of gout [3,4]	5.3 Adult patients with a diagnosis of gout currently taking diuretics	<25%

*CVD*, Cardiovascular disease; *eGFR*, Estimated glomerular filtration rate; *SUA*, Serum uric acid.

### Inclusion criteria

All adult patients currently registered with the practice who had a Read-coded diagnosis of gout in their medical record were eligible for inclusion in the audit. Read codes form a hierarchical system for recording morbidity types as well as symptoms, processes of care, and administrative information, and are commonly used as the basis of consultation recording in UK general practice.

### Search strategy

This audit was undertaken at one research-ready [11], advanced training primary care medical practice in North Staffordshire on 19^th^ July 2012. An electronic record search (INPS Vision version 3) using the Read codes “Of type C34..00 Gout” and “Of type N023.00 Gouty arthritis” was undertaken to identify patients with a current or past diagnosis of gout. All patients identified by the search who had related diagnoses, for example asymptomatic hyperuricaemia or uric acid renal stones, but no history of gout were excluded.

The electronic records of all relevant patients were searched to identify those who had a record of SUA (search “Uric acid blood level”), serum glucose (search “blood glucose”, “fasting glucose”), renal function (search “serum electrolytes”, “urea and electrolytes”, “glomerular filtration rate”), cardiovascular disease (CVD) risk assessment (search “scoring test result”), and ever use of ULT, diuretics and lipid lowering therapy (search drug class “long term control of gout”, “diuretic”, “lipid regulating drugs”).

### Data extraction and analysis

Medical records were reviewed by EC and VC. Data were extracted using a standard proforma, including information on demographic details such as gender and date of birth, gout-related information (date of diagnosis, prescription of ULT, SUA levels), documented CVD risk assessment and management, recommended lifestyle changes, and prescription of diuretics and lipid lowering therapy. Date of diagnosis was defined as the date of the first recorded entry of a Read code pertaining to gout. In instances where only the year was recorded, date of diagnosis was set to 30^th^ June of that year. 'Current’ medication use was defined as that which had been prescribed in the previous two months.

Data were analysed according to the criteria and standards, defined in Table 1. Descriptive analysis was undertaken using IBM SPSS Statistics version 20. Chi squared test was used to compare the proportion of people who had an eGFR recorded between those taking ULT and those who were not.

## Results

The total ≥18 yr practice population was 8686. Of these, 312 patients had an electronic diagnosis of gout, of whom seven had renal stones only, therefore the records of 305 (3.5%) patients with gout were analysed (see accompanying Additional file 1). Patient characteristics are summarised in Table 2.

**Table 2:** Characteristics of patients with gout

**Characteristic**	
Mean age (median, IQR)	65.5 years (67.0 years, 54.5-77.0)
Gender male, n(%)	227 (74%)
Mean duration since first electronic diagnosis of gout (median, IQR)	8.5 years (6.4 years, 1.4-13.0)
Proportion currently taking ULT, n(%)	77 (25%)

### Assessment of all gout patients

None of the standards relating to assessment of gout patients were reached. Among the 305 patients with gout, 226 (74%) had ever had a recorded SUA, 34 (11%) had ever had a recorded eGFR and 162 (53%) had ever had a recorded serum glucose since diagnosis (see Table 3). There was no significant difference in the proportion of patients who had ever had a recorded eGFR between those taking ULT (13%) and those who were not (11%, p = 0.55).

**Table 3:** Attainment of standards relating to assessment of gout patients

**Criterion**	**Standard**	**Attainment (n = 305)**	
1.1 Adult patients with a diagnosis of gout have ever had a recorded SUA	≥90%	226 (74%)	
1.2 Adult patients with a diagnosis of gout have ever had a recorded eGFR	≥90%	34 (11%)	
1.3 Adult patients with a diagnosis of gout have had a recorded serum glucose since diagnosis	≥90%	162 (53%)	
2.1 Adult patients currently prescribed (prescription in the last 2 months) allopurinol have a SUA in the last year	≥90%	26 (34%)	
2.2 Adult patients currently prescribed (prescription in the last 2 months) allopurinol have an SUA of ≤360 μmol/l	≥80%	29 (38%)	
		See Figure 1 and Figure 2	
3.1 Adult patients with a diagnosis of gout have a SUA in the last year	≥90%	68 (22%)	
3.2 Adult patients with a diagnosis of gout have had an eGFR in the last year	≥90%	27 (9%)	
4.1 Adult patients with a diagnosis of gout with evidence of advice regarding diet recorded	>50%	Diet discussed	43 (14%)
4.2 Adult patients with a diagnosis of gout with evidence of advice regarding alcohol recorded	>50%	Alcohol intake discussed	18 (6%)
		Alcohol type discussed	9 (3%)
		Advised about alcohol intake	17 (6%)
4.3 Adult patients with a diagnosis of gout with evidence of advice regarding fluid intake recorded	>50%	Maintain fluid intake 2 L/day	42 (14%)
4.4 Adult patients with a diagnosis of gout have a record of BMI	≥90%	BMI documented	304 (100%)
--	--	'Lifestyle advice’ documented	7 (2%)
--	--	PIL given	11 (4%)
5.1 Adult patients with a diagnosis of gout have a recorded CVD risk assessment score	≥90%	305	105 (34%)
5.2 Adult patients with a diagnosis of gout and a 10 yr risk of CVD ≥20% are prescribed lipid lowering therapy	≥75%	56	30 (54%)
5.3 Adult patients with a diagnosis of gout currently taking diuretics	<25%	73 (24%)	

*BMI*, Body mass index; *PIL*, Patient information leaflet.

### Management of recurrent gout

One-hundred and five patients (34%) had ever used ULT. Seventy seven patients (25%) were 'currently prescribed’ ULT, of whom 76 (99%) were taking allopurinol and one was taking probenecid. Twenty six patients had previously been prescribed allopurinol, one had been prescribed febuxostat and another probenecid.

Of the 76 people currently taking allopurinol, the diagnosis of gout was first recorded on the same day that they were first prescribed ULT in 15 (20%). Among the 61 remaining patients currently taking allopurinol, 60 were prescribed this medication after the recorded diagnosis of gout (range 16 to 6527 days) and for one patient the gout diagnosis was coded 2201 days after initial allopurinol prescription.

Starting doses of allopurinol were 100 mg (62%), 200 mg (6%) and 300 mg (32%). Dose adjustment had occurred between none and five times since treatment initiation. Most patients (n = 58) remained on the starting dose and, of these patients, 19 (33%) had an SUA in range. Six patients (8%) currently taking allopurinol had no SUA measurements recorded.

**Figure 1:**
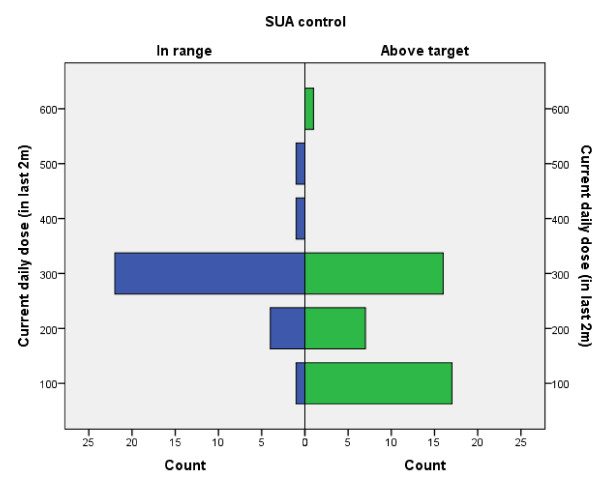
Number of patients with SUA in range (≤360 μmol/l) and above target among current users of allopurinol according to current daily dose.

**Figure 2:**
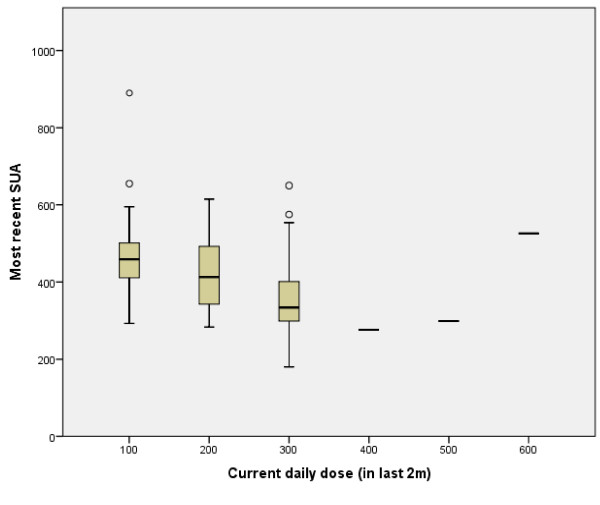
SUA values according to current allopurinol dose.

Only three patients were taking daily doses of allopurinol above 300 mg. The maximum dose taken by any patient was 600 mg. Control of SUA according to allopurinol dose is demonstrated in Figures 1 and 2. Amongst previous users of allopurinol (n = 26), 300 mg was the most common daily dose (n = 11).

### Monitoring patients with gout

Of the 305 patients with gout, 68 (22%) had a SUA level and 27 (9%) had an eGFR recorded in the last year. The proportion who had had an eGFR recorded in the last year did not differ between those who were taking ULT and those who were not (12% vs 8%, p = 0.31). Of the 76 patients currently taking allopurinol, 26 (34%) had a SUA level recorded in the last year and 29 (38%) had an SUA ≤360 μmol/l (see Table 3).

### Lifestyle advice

Although 304 patients with gout had a recorded BMI, recording of provision of other lifestyle advice was less frequent. There was evidence of discussion about diet (n = 43; 14%), fluid intake (n = 42; 14%), alcohol intake (n = 18; 6%) and alcohol type (n = 9; 3%). General entries pertaining to provision of lifestyle advice were identified by noting records that contained documentation of provision of patient information leaflets (n = 11; 4%) and “lifestyle advice” (n =7; 2%) (see Table 3), such entries were not mutually exclusive.

### Addressing comorbidities

**Figure 3:**
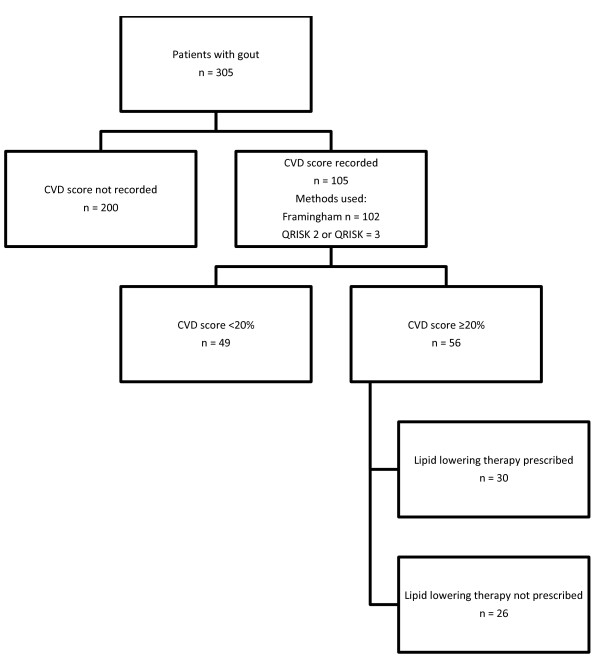
Management of CVD risk factors.

One hundred and five (34%) patients with gout had a recorded CVD risk assessment score. Of those with a recorded ten year score ≥20% (n = 56), 30 (54%) were receiving prescriptions for lipid lowering therapy (see Figure 3). Seventy-three (24%) patients with a diagnosis of gout were currently receiving a prescription for diuretics.

## Discussion and conclusions

Despite being the most prevalent form of inflammatory arthritis, gout is often poorly diagnosed and suboptimally managed [4]. Current research estimates that 1-2% of the UK adult population is affected by gout [9]. The prevalence of gout in the audited practice (≥18 years) was 3.5%. The results presented in this paper are important as they add to successive surveys which have shown persistent suboptimal management of gout in primary care. This audit is novel as it refers to both BSR/BHPR and EULAR guidelines and is the most comprehensive to date, covering a wider range of aspects of gout management than existing audits, namely: CVD risk factor screening, non-pharmacological management, use of ULT and 'treating-to-target’. New ways to encourage management and improve recording of care in line with best evidence is therefore needed and should be sought with some urgency.

### Assessment of all gout patients

The results demonstrated that 74% of patients with gout had a record of SUA. Although not a pre-requisite for diagnosis, a raised SUA supports a diagnosis of gout and measurement is essential if ULT is to be initiated and adequately titrated. Further, although gout with normouricaemia is well-recognised [12], persistently low SUA may necessitate reconsideration of the diagnosis.

Gout is associated with a number of comorbidities, the management of which may affect not only the development of gout but also the choice of therapeutic agent. Renal impairment and diabetes are notable thus current guidance recommends that patients have a record of both renal function and serum glucose after diagnosis [3]. Only 11% of patients with gout had an electronic record of eGFR and 53% a record of serum glucose. More frequent screening was found in an audit of twelve practices in 2000, which revealed that 66% of patients had a biochemical profile prior to initiation of treatment [8].

Of the 76 patients taking allopurinol, 26 patients (34%) had an SUA measurement recorded in the last year. Six patients (8%) currently taking allopurinol had no SUA measurements recorded, which is remarkable considering titration of allopurinol should occur according to SUA level.

### Management of recurrent gout

Allopurinol has been the mainstay of recurrent gout prevention for decades. The aim of such therapy is to reduce the SUA below the saturation point for monosodium urate thereby preventing crystal formation and deposition within the joints [4]. A target for a SUA of ≤360 μmol/l is set for patients on ULT. Roddy et al. found that persistent hyperuricaemia and recurrent acute attacks are still seen in patients taking allopurinol and suggest that doses >300 mg are often required [9].

Of the 305 patients identified as having gout, 105 (34%) were found to have ever used ULT. Of those currently taking allopurinol, 20% were prescribed ULT on the same day as the diagnosis was first formally coded. It is not clear from the medical records whether the date of diagnosis pertains to first attack or whether the patient was given additional, non-documented instructions as to how and when to take the medication prescribed. Historically, immediate prescriptions of allopurinol during an acute attack have been actively discouraged for fear of precipitating further acute flares. Although, recent evidence has started to suggest this approach may be unnecessary [13], advice about initiation should be documented.

Potentially correctable persistent hyperuricaemia increases the risk of recurrent gout flares and there is also an association with increased cardiovascular morbidity [14,15]. Current guidance regarding the initiation of allopurinol recommends a starting dose of 100 mg daily, with incremental increase of 100 mg every few weeks until the target level for SUA is achieved [3,4]. Only 62% patients were started on allopurinol at a dose recommended in the guidelines. Of those 102 patients ever prescribed allopurinol, the majority (57%) remained on the starting dose for the duration of therapy. When SUA among patients prescribed allopurinol was examined, only 34% of patients on ULT had a SUA recorded in the last year and only 29 (38%) patients had their latest SUA ≤360 μmol/l. These findings suggest that active titration of allopurinol according to SUA measurement in line with expert consensus [3] is not occurring in most patients, something that is not unique to this practice [16]. Wall et al. [10] also found that monitoring and dose titration against SUA was suboptimal, only 29% of patients in a suburban clinic having evidence of annual SUA measurement (average SUA ≈ 430 μmol/l among urban clinic and ≈ 410 μmol/l among patients in the suburban clinic). Roddy et al. [9] also found that only 9% of patients were taking allopurinol doses above 300 mg daily but 23% of patients taking allopurinol had SUA >360 μmol/l.

### Lifestyle advice

Provision of lifestyle advice appeared infrequent, echoing the findings of Pal et al. and Roddy et al. [8,9], although it is possible that this reflects poor documentation, rather than inadequate provision. However, this finding is not unique to gout with dietary advice documented in the primary care record of 43% patients at high risk of CVD and weight loss in 66% [17]. Historically there was no research data demonstrating a positive effect of education on gout and its treatment [4], however there was a general agreement that education does improve outcome. A recent study of patients referred to rheumatology outpatients has demonstrated that provision of information and education about the disease, its risk factors, clinical consequences and management strategies by a rheumatologist with specialist nurse follow up (telephone or in person) could result in 92% of patients achieving a SUA ≤360 μmol/l, with an average allopurinol dose of 400 mg daily and a reduction in pain at twelve months [18]. There remains a need to identify if such improvements can be gained from a primary-care based education approach and in a more general primary care population [17].

### Addressing comorbidities

Gout is associated with a number of comorbidities including metabolic syndrome, CVD, hypertension, and diabetes [2,19].

Of the 305 patients with gout, 73 patients (24%) were identified as taking at least one diuretic. This is similar to previous published findings which have identified the proportion of patients with gout taking diuretics ranging from 15-34% [2,8-10]. Although it is recognised that recent research evidence has not consistently demonstrated a trend towards a higher risk of acute gout in those patients receiving diuretic therapy [20], if diuretics are thought to be a precipitating or perpetuating factor through their effects on SUA, novel approaches to reducing such prescriptions may be required [4].

This audit looked for evidence of assessment of CVD risk. Indeed, both EULAR and BSR advocate CVD screening and subsequent treatment of risk factors in patients with gout [3,4]. Roddy et al. found that 31% of patients presenting with acute gout had undergone comorbidity screening within the 12 months prior to index consultation [21]. Similar suboptimal adherence to current recommendations was observed in the current practice as an electronic record of CVD risk assessment was found in only 34% of patients, of which 53% had a 10 yr CVD risk assessment score of ≥20%. Although those at higher risk may have a differentially high level of recording due to targeted intervention by clinicians, rigorous CVD risk assessment among the entire gout population may prove fruitful in detecting patients who stand to benefit from primary prevention methods.

### Limitations

Data collection relied on the presence of a Read-coded diagnosis of gout in the medical record. Although the high prevalence in this audit suggests that omission of substantial numbers of patients with gout is unlikely (e.g. those who have been given a joint symptom code), the methods do not exclude patients with an erroneously recorded diagnosis. Primary care consultation records have been widely used in gout research. In one such study, undertaken using consultation data, scrutiny of consultation free text recorded by GPs confirmed features of inflammation and distribution of affected joints generally consistent with a diagnosis of gout [21].

The duration of disease in people with gout has not been accurately detected by this audit. Analysis of the free-text associated with the coded diagnosis indicated that at least 15% probably had prior uncoded episodes. Pal et al. searched for people with diagnosis of gout and those on treatment for gout [8]. Such a method might have a greater sensitivity for detection of gout cases.

We found that 20% of patients currently taking allopurinol were recorded as being diagnosed with gout on the same day that they were first prescribed this ULT, demonstrating that either the diagnosis was made or coded retrospectively, or that management guidelines are not being followed.

As data was obtained from electronic records alone, it is impossible to estimate the frequencies with which episodes occurred for which patients did not consult. Thus adherence to guidelines regarding ULT initiation could not reliably be determined nor could the appropriateness of the timings of SUA measurements.

Current’ prescriptions were defined as those in which a prescription had been created in the two months preceding the audit search date. This method may over- or underestimate actual medication use. A more accurate picture may be obtained through conversion to defined daily doses.

This paper represents work from only one location, thus patient numbers are relatively small and will have limited direct generalisability. However, the findings from this audit do not differ greatly from published results of other audits and studies, indicating that the management of gout has not improved over the past decade. Data regarding ethnicity was not collected for this audit. The Office for National Statistics 2011 data suggests that the source population has a low representation of ethnic minority groups compared to the national picture [22].

This audit did not examine co-prescription of prophylactic medication at initiation of ULT to prevent acute flares. Although the importance of this is recognised, non-steroidal anti-inflammatory drugs are available over the counter thus actual patient use cannot be accurately quantified and record review may lead to under-estimation.

### Recommendations

A survey of UK General Practitioners in 2002 revealed that an overwhelming majority (86%) claimed to be confident in both the diagnosis and the management of gout [23]. However, the results of this audit are amongst many that have found that management of gout to be suboptimal, demonstrating poor concordance with current recommendations and guidelines [4,8,10,15,24,25] leading to many patients having SUA levels above target. Since this audit was undertaken, the American College of Rheumatology (ACR) have also published guidelines on the management of gout [26,27]. These newest guidelines continue to recommend patient education on diet, lifestyle and management of comorbidities as core features of gout management, describe allopurinol (or febuxostat) as first-line for lowering SUA and support previous allopurinol initiation and titration strategies [26]. The ACR guidelines also include additional recommendations such as more formalised consideration of causes of hyperuricemia, specific subgroup screening and combination or single novel pharmacological strategies. If we are to improve our management of gout through consideration of recent evidence-based recommendations, it is imperative that we first address deficiencies in the long-standing basics of management. Based on the findings of this audit and other work published for over a decade, several recommendations are made to facilitate improvement in the quality of care of patients with gout thereby reducing morbidity and associated CVD mortality and ultimately reduce the burden of disease.

#### ***Education of the general public***

Through raising public awareness of gout and the potential implications and links to other aspects of ill-health, it may be possible to modify health-seeking behaviour, thus encouraging presentation to primary care. Patient education may improve compliance with therapy, lifestyle modification and motivation to change, especially since the popular stereotypes of patients with gout are outdated and inaccurate.

#### ***GP continued professional development for the diagnosis and management of gout***

Before GPs will adhere to recommendations in guidelines, they need to first be aware of them [28]. Strategies to increase awareness must include improved, accessible dissemination and promotion of the key recommendations from current guidelines. The use of routine quality indicators of care [29] may also be beneficial to prompt GPs to consider best evidence-based care. Ideally patients should be involved in the development of quality indicators to ensure they address priority areas, and take into account the preferences of, the target population [30]. Education regarding the importance of gout as a risk factor for cardiovascular disease and the importance of screening for modifiable risk factors may provide further impetus to improve care. Locally the authors acted on the audit findings in the practice by outlining the guideline recommendations, highlighting current deficiencies in records of care and arranging for a local expert to give a talk to the practice team. More widely, presentation of audit findings at academic and clinical meetings will raise awareness and prompt other professionals to reflect on their own practice. However, there remains a need to find effective ways of nationally raising awareness of relevant recommendations across primary care.

#### ***Widen guideline development groups to involve patients and primary care professionals***

Once professionals are aware of guidelines, they must agree with the contents before they will adopt them [28]. Involving primary healthcare professionals in guideline development for the management of gout will ensure that recommendations are relevant and deliverable within primary care. Further, through adequate representation of patients in guideline development groups, guidelines that address patient’s priorities, taking into account their lived experiences of the condition, will also be developed. Although the Arthritis and Musculoskeletal Alliance (ARMA) standards for the management of gout [31] included primary care representatives, neither the EULAR nor BSR/BHPR guidelines, which provided the standards for this audit, included a GP, nor did they clearly involve a patient, in their development groups.

#### ***Consider financially incentivising gout care or demonstrating pre-existing related targets***

Gout is not currently part of the UK payment-for-performance scheme, the Quality and Outcomes Framework (QOF). Maisey et al. have previously found that whilst substantial improvement in the quality of care of conditions included in QOF was observed, the same was not so for other non-incentivised conditions [32]. It is possible that incentivising care may increase both awareness and adoption of best evidence guidelines.

#### ***Educate healthcare professionals and administrative staff about the importance of specific and accurate coding***

It is known that diagnostic coding of acute conditions is not always accurate [33]. The accuracy and consistency with which coding is performed could be improved by educating healthcare professionals and administrative staff about the importance of and/or encouraging use of specific and accurate coding to promote continuity of care, optimise management through a systematic annual recall system and allow for accurate routine audit and epidemiological research.

#### ***Reorganising gout management in primary care***

The final step in providing best evidence care from guidelines is to adhere to the guidelines [28]. Adherence may be encouraged through the use of prompts triggered by disease-specific Read codes during individual consultations. Integration of clinical templates, to ensure that appropriate advice, drug titration, review of existing medications and investigations are undertaken may address some deficiencies. Such a template-based system could be triggered by prescription or medication review of allopurinol, and could facilitate practice nurse follow-up and dose titration once patients have been counselled about and commenced on allopurinol by their GP.

#### ***Future research***

To have a major driver for change in the management of gout, further work to understand the link between gout and CVD is needed. In particular, the benefits of controlling hyperuricaemia and any reduction in concomitant CVD risk should be carefully assessed. This novel approach to the risks associated with persistently elevated SUA would help both clinicians and patients to prioritise the management of gout in the context of individual and population morbidities. Further, qualitative investigation into the attitudes and beliefs of GPs and patients regarding the management of gout as well as awareness and experiences of implementing current guidelines would provide additional information that may highlight barriers to improving evidence based practice.

## Abbreviations

ACR: American College of Rheumatology; BHPR: British Health Professionals in Rheumatology; BMI: Body mass index; BSR: British Society for Rheumatology; CVD: Cardiovascular disease; EGFR: Estimated glomerular filtration rate; EULAR: European League Against Rheumatism; PIL: Patient information leaflet; QOF: Quality and outcomes framework; SUA: Serum uric acid; ULT: Urate-lowering therapy.

## Competing interests

EC and VC are GP registrars and JE is a principal in general practice at the audited practice. ER was a co-author of the EULAR recommendations.

## Authors’ contributions

EC and VC devised the original concept for the audit which was subsequently designed and developed with JE. EC and VC collected and analysed the data with support from JE. EC, VC, JE and ER were all involved in interpreting the data, drafting and revising the manuscript and all authors read and approved the final manuscript.

## Authors’ information

EC and VC were third year General Practice Specialty Trainees at the time the audit was undertaken. EC is an NIHR Academic Clinical Fellow at Keele University and VC is now a General Practitioner and is undertaking a Masters project in musculoskeletal medicine at Keele University.

JE is a principal in general practice and GP Research Fellow at Keele University.

ER is an Honorary Consultant Rheumatologist with specific expertise in gout and is Clinical Senior Lecturer in Rheumatology at Keele University.

## Pre-publication history

The pre-publication history for this paper can be accessed here:

http://www.biomedcentral.com/1471-2296/14/170/prepub

## Supplementary Material

Additional file 1 Identification of patient sample and measurement of serum uric acid among patients with gout. Click here for file
