# Supplementary material for: Improvement in the management of gout is vital and overdue: an audit from a UK primary care medical practice
Source: BMC Fam Pract. 2013 Nov 14;14:170. doi: 10.1186/1471-2296-14-170 (PMC3830984; doi:10.1186/1471-2296-14-170)
Supplement: Supplementary file 1 [file 1471-2296-14-170-S1.docx]

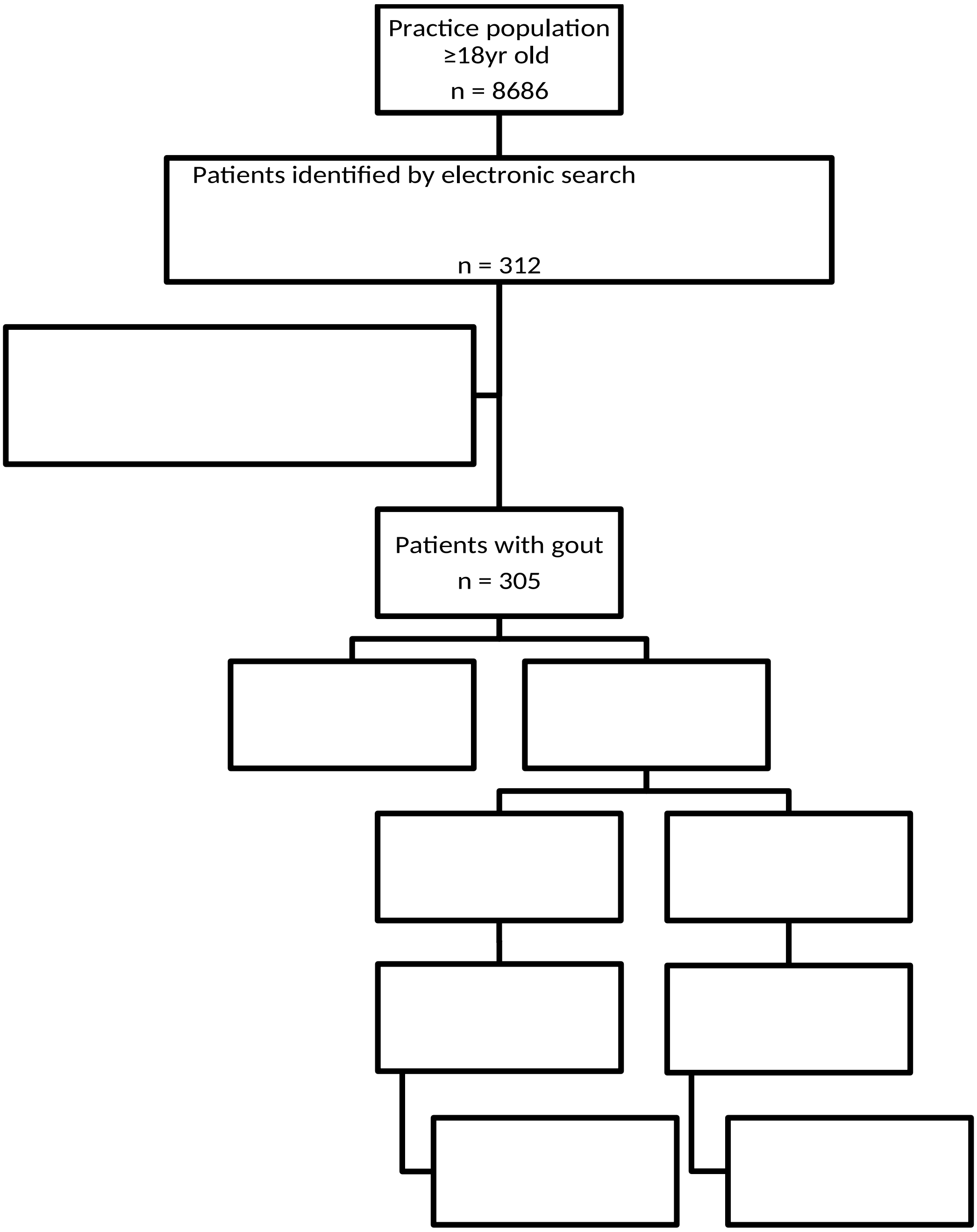
Practice population
≥18yr old
n = 8686
Patients identified by electronic search
n = 312
Patients with gout
n = 305
